# Chemical Composition and Biological Activities of Essential Oils of Four *Asarum* Species Growing in Vietnam

**DOI:** 10.3390/molecules28062580

**Published:** 2023-03-12

**Authors:** Pham Thi Hong Minh, Nguyen Thuong Tuan, Nguyen Thi Hong Van, Hoang Thi Bich, Do Tien Lam

**Affiliations:** 1 Institute of Natural Products Chemistry, Vietnam Academy of Science and Technology (VAST), 18 Hoang Quoc Viet, Cau Giay, Hanoi 10072, Vietnam; 2 Faculty of Chemistry, Graduate University of Science and Technology, VAST, 18 Hoang Quoc Viet, Cau Giay, Hanoi 10072, Vietnam; 3 Institute of Life Sciences, Thai Nguyen University of Agriculture and Forestry, Quyet Thang, Thai Nguyen 24119, Vietnam

**Keywords:** *Asarum*, *Asarum geophilum*, *Asarum yentunensis*, *Asarum splendens*, *Asarum cordifolium*, essential oil, antimicrobial, antioxidant

## Abstract

The essential oils (EOs) of the aerial parts of four *Asarum* species (*A. geophilum*, *A. yentunensis*, *A. splendens* and *A. cordifolium)* were isolated by steam distillation and analyzed by the GC/MS method. The *A. cordifolium* EO contains 33 constituents with the main component being elemicine (77.20%). The *A. geophilum* EO was contains 49 constituents with the main components being determined as 9-*epi*-(*E*)-caryophyllene (18.43%), eudesm-7(11)-en-4-ol (13.41%), *β*-caryophyllene (8.05%) and phytol (7.23%). The *A. yentunensis* EO contains 26 constituents with the main components being safrole (64.74%) and sesquicineole (15.34%). The EO of *A. splendens* contains 41 constituents with the main components being 9-*epi*-(E)-caryophyllene (15.76%), eudesm-7(11)-en-4-ol (14.21%), *β*-caryophyllene (9.52%) and *trans*-bicyclogermacrene (7.50%). For antimicrobial activity, the *A. yentunensis* EO exhibited the highest inhibition activity against *Staphylococcus aureus* and the *A. cordifolium* EO against *Bacillus subtillis* (MIC values of 100 μg/mL). For antioxidant activity, the *A. geophilum* EO showed the highest potential with an SC (%) value of 63.34 ± 1.0%, corresponding to an SC_50_ value of 28.57 µg/mL. For anti-inflammatory activity, the *A. splendens* EO exhibited the highest potential with an IC_50_ value of 21.68 µg/mL, corresponding to an inhibition rate of NO production of 69.58 ± 1.3% and the percentage of cell life was 81.85 ± 0.9%.

## 1. Introduction

The *Asarum* genus belonging to the Aristolochiaceae family, including 100 species in the world, is distributed mainly in the Northern Hemisphere, East Asia from the Himalayas to China, Taiwan, Japan, South Korea, Sakhalin island, North America and Europe [1,2]. In Vietnam, there are eleven *Asarum* species, including *A. balansae, A. blumei, A. caudigerum, A. glabrum, A. petelotii, A. reticulatum, A. wulingense*, *A. geophilum, A. yentunensis, A. splendens,* and *A. cordifolium* distributed mainly in the North of Vietnam, except for *A. wulingense* which was discovered in Central Vietnam in Ha Tinh province [3]. Four *Asarum* species were used in traditional medicine *A. geophilum, A. yentunensis, A. splendens,* and *A. cordifolium* [3,4].

Plants of the genus *Asarum* are rich in EOs which are the components possessing biologically or pharmacologically active ingredients. Up to now, more than 155 compounds have been identified from the EO of genus *Asarum*, whose main characteristic components are phenylpropanoids (elemicin, safrole, asarone, methyleugenol, myristicin, eugenol, etc.), terpenoid derivatives and aromatic compounds. The higher content of methyleugenol, kakuol, myristicin, asarone, elemicin, eucarvone, sesamin, asarinin, etc., may be the main toxic ingredients when used in high doses [5,6,7].

The *A. heterotropoides* var. *mandshuricum* EO was found to have antibacterial, anti-phytopathogenic, antidepressant effects and fumigant toxicity. In detail, the *A. heterotropoides* EO showed antimicrobial effect towards five types of epidermal bacteria producing human body odor *Corynebacterium jeikeium, Corynebacterium xerosis, Micrococcus luteus, Propionibacterium freudenreichii,* and *Staphylococcus epidermidis,* with MIC values ranging from 10.1 to 46 mg/mL [8]; against three periodontal pathogens *Porphyromonas gingivalis, Prevotella intermedia* and *Fusobacterium nucleatum*, in vitro and in vivo [9]; inhibited five pathogenic fungi infecting plants *Alternaria humicola, Colletotrichum gloeosporioides, Rhizoctonia solani, Phytophthora cactorum* and *Fusarium solani* with IC_50_ values < 0.42 μg/mL [10]; fumigant toxicity to *Tetranychus urticae*, at a fumigant concentration of 8 μg/mL; the mortality rate of mites was 72.6% in 24 h, and reached 100% after 48 h [11]. This essential oil also exhibited an inhibitory effect on the growth and development of *Streptoccus* sp., *Shigella* sp. and *Salmonella typhi* [12].

The *A. sieboldii* EO exhibited strong insecticidal activity against *Sitophilus oryzae* with an LC_50_ of 2.37 μg/mL; eucarvone and safrole were the most effective compounds, with LC_50_ values of 3.32 and 11.27 μg/mL [13]. For individual compounds in volatile oil, methyleugenol, *β*-asarone and myristicin showed significant insecticidal activity against the tobacco beetle *Lasioderma serricorne*, with LC_50_ values of 720, 60, and 540 ppm [14]. The *A. sieboldii* EO demonstrated very high antifungal activity against the *Aspergillus fumigatus, Aspergillus niger, Cryptococcus neoformans*, *Candida albican* and *Neolentinus lepideus* [2,15].

The compounds elemicin and (N-Isobutyl-(2E,4Z,8Z,10E)-dodecatetraenamide isolated from the genus *Asarum* EOs noticed a significant anti-allergic activity by the inhibitory effect on enzyme 5-lipoxygenase with IC_50_ values of 6.0 μM and 0.16 μM [16].

The chemical composition of EOs of some *Asarum* species growing in Vietnam has been studied. The major constituent of the trunk and leaf EO of *A. caudigerum* was safrol (96.2%) [17], while the major constituents of the trunk and leaf EO of *A. glabrum* were safrol (42.24%), apiole (27.11%) and myristicin (6.13%) [18]. Elimicin was the major compound of *A. balansae* and *A. cordifolium* EO (71.53% and 84.38%, respectively), while *E*-methyl isoeugenol and myristicine were major compounds of *A*. *yunnanense* and *A. petelotii* EO (47.39% and 59.06%, respectively) [19]. The major constituent of the leaf EO of *A. geophyllum* was 9-epi-*β*-caryophyllene (27.16%), bicyclogermacrene (16.98%) and *β*-caryophyllene (11.91%) [17], while the major constituents of the roots and rhizomes EO of *A. geophyllum* were eudesm-7(11)-en-4-ol (16.94%), *β*-pinene (12.61%), aristolene (7.01%) and 9-epi-*β*-caryophyllene (7.88%) [20].

The aim of this study was to evaluate the chemical composition and antimicrobial, antioxidant, and anti-inflammatory activities of the aerial part essential oils of four *Asarum* species growing in Vietnam, including *A. geophilum, A. yentunensis, A. splendens* and *A. cordifolium*.

## 2. Results and Discussion

### 2.1. Chemical Composition of the Essential Oils

The EOs of the aerial parts (leaves and stems) from four *Asarum* species (*A. geophilum, A. yentunensis*, *A. splendens* and *A. cordifolium*) were obtained by steam distillation in a Clavenger apparatus for 7 h. The chemical composition of these EOs was analyzed by gas chromatography coupled with mass spectrometry (GC-MS) (Table 1).

**Figure 1:**
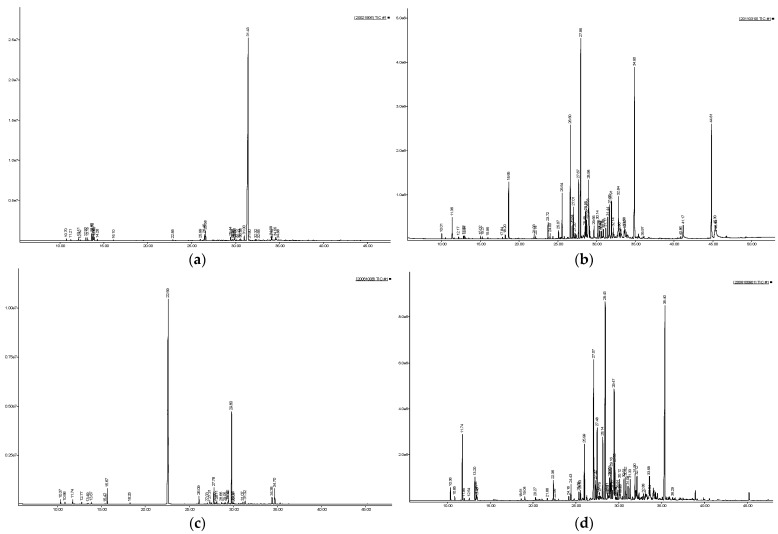
The GC-MS spectrums of the EOs of four *Asarum* species. (**a**) *Asarum cordifolium*, (**b**) *Asarum geophilum,* (**c**) *Asarum yentunensis,* (**d**) *Asarum splendens*.

For the *A. cordifolium* EO with a yield of 0.30% (*w*/*w*, based on fresh material), 33 compounds were identified, accounting for 98.78%, of which there were ten monoterpene hydrocarbons (7.58%), two oxygenated monoterpenes (0.43%), eleven sesquiterpene hydrocarbons (6.53%), five oxygenated sesquiterpenes (1.96%) and five derivatives of benzene (benzenoids) (82.03%). The main component of this EO was elemicin (77.20%) (Figure 1a, Table 1). This result was similar to the reference reported that elemicin presents with a high amount in the *A. cordifolium* EO (84.38%) [19]. Elemicin has been also previously found in EOs of other *Asarum* species: *A. sieboldii* (4.8–11.1%); *A. himalaicum* (13.1–42.2%), *A. canadense* (4.9%), and *A. insigne* (5.4%) [2,5]. Elemicin has been known to be the main ingredient that leads to the antibacterial and antifungal activities of some essential oils [2]. The EO of *Daucus carota* L. spp. *carota* (16.3% elemicin) was active against *Campylobacte jejuni*, *Campylobacte coli*, and *Campylobacte lari* [21]. The EO of *Daucus carota* subsp. *halophilus* (26.0% elemicin) was also shown to have antimicrobial activity with MIC values ranging from 0.16 to 0.32 µL/mL [22].

For the EO of *A. geophilum,* with a yield of 0.19% (*w*/*w*, based on fresh material), 49 compounds were identified, accounting for 93.05%. There were five monoterpene hydrocarbons (2.46%), seven oxygenated monoterpenes (5.41%), seven-teen sesquiterpene hydrocarbons (46.84%), eleven oxygenated sesquiterpenes (23.29%), one benzenoid (0.17%) and eight other constituents (14.88%). The main components were 9-*epi*-(*E*)-caryophyllene (18.43%), eudesm-7(11)-en-4-ol (13.41%), *β*-caryophyllene (8.05%), phytol (7.23%), *cis*-bicyclogermacrene (4.46%) and *α*-terpineol (4.07%) (Figure 1b, Table 1). This result was similar to the reference that reported that eudesm-7(11)-en-4-ol and *β*-caryophyllene are present in high amounts in the *A. geophilum* EO [20]. However, there was a significant difference of 9-*epi*-(E)-caryophyllene (18.43%) with a high amount in the studied *A. geophilum* EO and a low amount in the reference reported and vice versa, a high amount of *β*-pinene (12.61%) in the reference reported and low amount in the studied *A. geophilum* EO [20]. The components and contents of the *A. geophilum* EO were different, maybe due to differences in the geographical locations, soil and harvesting conditions.

For the EO of *A. yentunensis,* with a yield of 0.05% (*w*/*w*, based on fresh material), 26 compounds were identified, accounting for 96.30%. There were seven monoterpene hydrocarbons (3.21%), two oxygenated monoterpenes (3.50%), nine sesquiterpene hydrocarbons (6.00%), two oxygenated sesquiterpenes (15.75%) and six benzenoids (67.84%). The main components of this EO were safrole (64.74%), sesquicineole (15.34%), linalool (3.19%) and (*Z*)-*β*-farnesene (2.81%) (Figure 1c, Table 1). Safrole has been also known to be a main component of the EO of other *Asarum* species: *A. caudigerum* (96.2%) [17] and *A. glabrum* (42.24–46.60%) [19,23].

For the EO of *A. splendens*, with a yield of 0.11% (*w*/*w*, based on fresh material), 41 compounds were identified, accounting for 91.36%, including seven monoterpene hydrocarbons (7.01%), eight oxygenated monoterpenes (3.45%), twenty-five sesquiterpene hydrocarbons (59.06%), seven oxygenated sesquiterpenes (19.13%), one benzenoid (1.49%) and three others compounds (1.49%). The main components of this EO were 9-*epi*-(*E*)-caryophyllene (15.76%), eudesm-7(11)-en-4-ol (14.21%), *β*-caryophyllene (9.52%), *trans*-bicyclogermacrene (7.50%), *β*-pinene (4.71%) and *cis-β*-elemene (3.48%) (Figure 1d, Table 1). This result shows that the EO of *A. cordifolium* has sesquiterpene hydrocarbons (25/41 components, 59.06%) higher than other *Asarum* species [4].

The constituents: *α*-pinene (0.33–0.84%), *β*-pinene (0.91–4.71%) and (*E*)-nerolidol (0.32–0.79%) were present in the four species studied.

Three components *α*-pinene (0.33–0.84%), *β*-pinene (0.91–4.71%) and (*E*)-nerolidol (0.32–0.79%) are present in all four sudied *Asarum* species. In the EOs of *A. geophilum* and *A. splendens*, sesquiterpene hydrocarbons and oxygenated sesquiterpenes were dominant constituents: 17/49 components with 46.84% and 11/49 components with 23.29% for *A. geophilum* EO; 25/41 components with 59.06% and 7/41 components with 19.13% for *A. splendens* EO. Meanwhile, in the essential oils of *A. yentunensis* and *A. cordifolium*, benzenoids were dominant constituents: 6/26 components with 67.84% for *A. yentunensis* EO and 5/33 components with 82.03% for *A. cordifolium* EO; monoterpene hydrocarbons and sesquiterpene hydrocarbons were also present in this two EOs but with a quantity lower than that of *A. geophilum* and *A. splendens* EOs (7/26 components with 3.21% and 9/26 components with 6.0% for *A. yentunensis* EO; 10/33 components with 7.58% and 11/33 components with 6.53% for *A. cordifolium* EO.

Methyl ether (0.13%), geranial (0.18%), *endo*-isocamphanyl acetate (1.01%), geranyl acetate (0.51%), γ-muurolene (0.59%), *ar*-curcumene (0.63%), *trans*-muurola-4(14),5-diene (0.94%), *β*-bisabolene (1.65%), cuparene (0.16%), zonarene (0.24%), humulene epoxide II (0.14%) and (*Z*)-ligustilide (0.48%) were common components for the four *Asarum* EOs. However, *exo*-fenchol, *α*-terpinyl acetate (0.35%), viridiflorene (1.48%), elemol (0.41%), 4-*epi*-maaliol (0.67%), viridiflorol (2.16%), cubeban-11-ol (2.27%), rosifoliol (0.62%), *neo*-intermedeol (1.01%), *n*-tetradecanoic acid (0.18%), isophytol (0.12%), *n-*hexadecanoic acid (1.31%), phytol (7.23%), linoleic acid (2.45%) and linolenic acid (2.31%) were characteristic for the *A. geophilum* EO*;* while safrole (64.74%), (*E*)-*β*-farnesene (0.19%), *γ*-curcumene (0.24%), (*E*)-methyl isoeugenol (0.27%), (*E,E*)-*α*-farnesene (0.36%), sesquicineole (15.34%), myristicin (0.31%), *β*-sesquiphellandrene (0.16%) and *iso*elemicin (0.32%) were characteristic for the *A. yentunensis* EO; *exo*-fenchol, *δ*-selinene (0.17%), *γ*-amorphene (0.33%), *α*-bisabolene (1.18%), elemicine (77.20%), scapanol (0.33%), *α*-asarone (0.73%) and *α*-murolol (0.24%) were characteristic for the *A. cordifolium* EO.

Safrole [24], elemicin [21], 9-*epi*-(*E*)-caryophyllene [25] and eudesm-7(11)-en-4-ol [26] showed moderate antimicrobial, antioxidant and anti-inflammatory activity. The difference in chemical composition maybe led to the difference in biological activities of these four *Asarum* EOs.

### 2.2. Antimicrobial Activity of the EOs

The biological activity of the EOs of the aerial parts of *A. geophilum, A. yentunensis, A. splendens* and *A. cordifolium* were evaluated in terms of anti-microbial activity on eight strains of fungi, yeast, and bacteria (Table 2).

The results showed that the *A. geophilum* EO exhibited good inhibition activity on the *Escherichia coli, Pseudomonas aeruginosa*, *Fusarium oxysporum* and *Saccharomyces cerevisiae* with a MIC value of 200 μg/mL. The *A. yentunensis* EO demonstrated potent inhibition activity on the *Escherichia coli, Aspergillus niger* and *Candida albicans* with a MIC value of 200 μg/mL, and on the *Staphylococcus aureus* with a MIC value of 100 μg/mL. The *A. splendens* EO exhibited moderate inhibition activity on the *Escherichia coli, Saccharomyces cerevisiae* and *Fusarium oxysporum* with a MIC value of 200 μg/mL. The *A. cordifolium* EO exhibited potent inhibition activity on the *Escherichia coli, Bacillus subtillis* and *Candida albicans* with a MIC value of 200 μg/mL, and on the *Bacillus subtillis* with a MIC value of 100 μg/mL.

For the antibacterial activity against Escherichia coli; Pseudomonas aeruginosa, Bacillus subtillis and Staphylococcus aureus showed:

*Escherichia coli* is a Gram-negative, a large and diverse group of bacteria, found in the lower intestine of people and animals. Some kinds of *Escherichia coli* can make you sick and cause diarrhea, cause urinary tract infections, respiratory illness and pneumonia, etc. [27,28]. All four species *Asarum* EOs demonstrated moderate antibacterial activity with a MIC value of 200 μg/mL. This suggests the potential for the *Asarum* EOs to have antimicrobial activity against growing *Escherichia coli.*

*Pseudomonas aeruginosa* is a Gram-negative, aerobic, extremely versatile, antibiotic-resistant bacteria and causes infections in the blood, lungs, or other parts after surgery [29]. Only *A. geophilum* EO showed activity against *Pseudomonas aeruginosa* with a MIC value of 200 μg/mL. Despite the high contents and predominant composition of the EOs from *A. geophilum* and *A. splendens* being similar, the *A. splendens* EO showed no activity. This may be due to the presence of alcohols and fatty acids (14.88%) in the *A. geophilum* EO.

*Bacillus subtillis* is a Gram-positive, ubiquitous bacteria, it is not pathogenic and produces important commercial products (fermented products, sweeteners, flavor enhancers and animal feed additive) [30,31]. Only *A. cordifolium* EO exhibited potent antibacterial activity against *Bacillus subtillis* with a MIC value of 100 μg/mL. Elimicine presents with a high amount in the *A. cordifolium* EO (77.20%).

*Staphylococcus aureus* is a Gram-positive bacteria and has a wide variety of clinical manifestations. Infections caused by this pathogen are common both in community-acquired and hospital-acquired settings [32,33]. The *A. yentunensis* EO with the main components: safrole (64.74%) and sesquicineole (15.34%) demonstrated stronger inhibition activity than the *A. splendens* EO with the main components: 9-*epi*-(*E*)-caryophyllene (15.76%), eudesm-7(11)-en-4-ol (14.21%) and *β*-caryophyllene (9.52%), corresponding to MIC values of 100 and 200 μg/mL. However, high safrole content is potentially phytotoxic [5].

For the antifungal activity against Aspergillus niger, Fusarium oxysporum, Saccharomyces cerevisiae and Candida albicans:

*Aspergillus niger* is among the most common fungi, responsible for post-harvest decay of fresh fruit, fish products and meat products [34,35]. Only the *A. yentunensis* EO with high amounts of safrole (64.74%) and sesquicineol (15.34%) was likely responsible for these antifungal activities.

*Fusarium oxysporum* is the common soilborne fungi and the fungal communities in the rhizosphere of plants. All strains of *Fusarium oxysporum* are saprophytic and penetrate into the roots inducing either root rots or tracheomycosis [36]. The EOs of *A. geophilum* and *A. splendens* were rich in contents of sesquiterpene hydrocarbons and oxygenated sesquiterpenes, and exhibited good inhibition activity against *Fusarium oxysporum* with MIC values of 200 μg/mL.

*Saccharomyces cerevisiae* is a species of budding yeast, responsible for bread formation and alcohol production. It is useful in studying the cell cycle [37]. Only *A. geophilum* EO showed antifungal activity with a MIC value of 200 μg/mL and may have been related to compounds of alcohol and fatty acid.

*Candida albicans* is a yeast that lives on the human body in small amounts and is responsible for infections such as thrush and vaginal yeast infections, etc. [38]. The *A. yentunensis* EO and *A. cordifolium* EO showed demonstrated inhibition activity against *Candida albicans* with MIC values of 200 μg/mL. The main components: safrole and elemicine could have been responsible for these antifungal activities. However, it needs to be studied further for its antifungal activity.

### 2.3. Antioxidant Activity of the EOs

The results of in vitro antioxidant activity testing by the DPPH method of the four *Asarum* EOs with the positive control as ascorbic acid were shown in Table 3.

The obtained results showed that the *A. geophilum* EO displayed the best antioxidant activity with an SC (%) value of 63.34 ± 1.0%, corresponding to the SC_50_ value of 28.57 µg/mL. The *A. cordifolium* EO with an SC_50_ value of 57.86 ± 0.8% corresponded to an SC_50_ value of 39.62 µg/mL and the *A. yentunensis* EO with an SC_50_ value of 51.58 ± 0.5% corresponded to SC_50_ value of 50.24 µg/mL. The *A. splendens* EO did not show activity at the tested concentration. These results have shown the good antioxidant capacity of *Asarum* EOs.

The *A. geophilum* EO displayed better antioxidant activity (SC_50_ value of 28.57 µg/mL) than the *A. splendens* EO (SC_50_ value of over 100 µg/mL). The EOs of *A. geophilum* and *A. splendens* were of similar high amounts of the predominant composition of sesquiterpene hydrocarbons and oxygenated sesquiterpenes. This difference may be due to the different content of alcohols and fatty acids in these two species (14.88% of in the *A. geophilum* EO and 1.49% of the *A. splendens* EO).

Although the EOs of *A. yentunensis* and *A. cordifolium* were the similar benzenoids dominant constituents (67.84% for *A. yentunensis* EO and 82.03% for *A. cordifolium* EO) the *A. cordifolium* EO showed the better antioxidant activity (SC_50_ value of 39.62 µg/mL) than the *A. yentunensis* EO (SC_50_ value of 50.24 µg/mL). The main component of *A. cordifolium* EO (elemicine: 77.20%) and *A. yentunensis* EO (safrole 64.74%) are interesting ways to explain this antioxidant activity results.

### 2.4. Anti-Inflammatory Activity of the EOs

The results of testing the in vitro anti-inflammatory activity of the four *Asarum* EOs were evaluated through the inhibition of NO production by using LPS- on RAW 264.7 as shown in Table 4.

The results showed that the *A. splendens* EO exhibited the best anti-inflammatory activity through inhibition of NO production with an IC_50_ value of 21.68 µg/mL. The next are *A. geophilum* EO with an IC_50_ value of 40.35 µg/mL and *A. yentunensis* EO with an IC_50_ value of 49.87 µg/mL. Finally, the *A. cordifolium* EO illustrates lower anti-inflammatory activity at the tested concentration with an IC_50_ value of 66.37 µg/mL.

The anti-inflammatory activity of the EOs rich in contents of benzenoids from *A. yentunensis* and *A. cordifolium* was better than the EOs rich in contents of sesquiterpene hydrocarbons and oxygenated sesquiterpenes from the *A. geophilum* and *A. splendens* with IC_50_ values from 21.68 to 40.35 µg/mL and from 49.87 to 66.37 µg/mL, respectively.

For the anti-inflammatory activity of the *A. splendens* EO, the main components of 9-*epi*-(*E*)-caryophyllene (15.76%), eudesm-7(11)-en-4-ol (14.21%), *β*-caryophyllene (9.52%) and *trans*-bicyclogermacrene (7.50%) exhibited the best anti-inflammatory activity through the inhibition of NO production with an IC_50_ value of 21.68 µg/mL, produced NO inhibition of 69.58 ± 1.3% and a high cell survival rate of 81.85 ± 0.9%. It has the potential to be developed into future anti-inflammatory drugs because of their effectiveness and safety.

The anti-inflammatory activity of the *A. geophilum* EO with 9-*epi*-(*E*)-caryophyllene (18.43%), eudesm-7(11)-en-4-ol (13.41%) and *β*-caryophyllene (8.05%) as the main components demonstrated moderate anti-inflammatory activity with an IC_50_ value of 40.35 µg/mL (inhibition rate of NO production was 58.20 ± 0.4% and the cell survival rate was 78.05 ± 0.8%). In China, the species *Asarum* produces pungent aromatic roots that are used in traditional medicine as a remedy for pain and colds. The above results have contributed to elucidating this activity in traditional Chinese medicine [2,5].

For the anti-inflammatory activity of the *A. yentunensis* EO, the main components of safrole (64.74%) and sesquicineole (15.34%) displayed the anti-inflammatory activity with IC_50_ value of 49.87 µg/mL (inhibition rate of NO production was 53.14 ± 1.6% and the cell survival rate was 66.87 ± 1.5%). In particular, the low cell survival rate may be due to the toxicity of safrole. *A. yentunensis* is an endemic species for the flora of Vietnam. Therefore, the study has clarified the chemical composition and biological activity of this species.

For the anti-inflammatory activity of the *A. cordifolium* EO with elemicin (77.20%) as the main component, a lower anti-inflammatory activity was found with an IC_50_ value of 21.68 µg/mL; NO inhibition only reached 40.87 ± 0.6% and cell survival rate of 60.65 ± 1.4%. The low cell survival rate may be due to the toxicity of elemicin. That contributes to explaining the meaning of the Red Dao Minority Vietnam (Sapa town, Lao Cai province) use in folk medicine to treat back pain and wound infections.

## 3. Materials and Methods

### 3.1. Plant Materials

The fresh aerial parts of four *Asarum* species (*Asarum geophilum*, *Asarum cordifolium*, *Asarum splendens*, *Asarum yentuense*) were collected and identified by Dr. Nguyen Anh Tuan, Indochina Institute of Biological and Environmental sciences and Dr. Nguyen Quoc Binh, Vietnam National Museum of Nature, VAST. The herbarium specimen was deposited at the Institute of Natural Products Chemistry, VAST. *Asarum geophilum* Hemsl. was collected in Trung Khanh district, Cao Bang province in May 2020. *Asarum cordifolium* C. E. C. Fischer and *Asarum splendens* (F.Maek.) C.Y.Chen and C.S.Yang were collected in Sapa district, Lao Cai province in January 2021 and June 2020, respectively. *Asarum yentuense* N. Tuan and Sasamoto was collected in Uong Bi district, Quang Ninh province in June 2020.

### 3.2. Extraction the EOs

The fresh aerial parts of four *Asarum* species (500 g/a species/a time) were washed with water, allowed to dry at room temperature, minced, put in a round-bottom flask 3 L, added to 1000 mL of pure water and subjected to hydrodistillation for 7 h using a Clevenger type apparatus. The obtained EOs were dehydrated with anhydrous Na_2_SO_4_, kept in sealed glass vials and stored at −15 °C [39,40]. The experiments of each species were repeated three times to determine the EOs contents compared with fresh samples. Then they were pooled for component determination by GC-MS analysis and bioactivity was tested in vitro.

### 3.3. Analyzing Chemical Constituents of the Essential Oils

The chemical constituents of the EOs of four species from genus *Asarum* were determined by combining the GC-FID and GC–MS system with the standard library and the MASSFinder library of natural compounds: The GC-MS analysis was carried out with Agilent Technologies HP7890A GC equipped with a mass spectrum detector (MSD) Agilent Technologies HP5975C, and an HP5-MS column (60 m × 0.25 mm, film thickness 0.25 µm). The GC-FID analysis was carried out with the same conditions as those for the GC-MS analysis. MassFinder 4.0 software connected to the HPCH1607, W09N08 libraries and the NIST Chemistry WebBook was used to match mass spectra and retention indices [41]. The analysis was conducted at the Chemical Analysis Lab, Institute of Natural Products Chemistry, The Vietnam Academy of Science and Technology.

### 3.4. Antimicrobial Assays

The method of assessment of antimicrobial activity was conducted to evaluate the antibiotic activities of EO samples by the method of McKane and Kandel (1996) [42,43] on eight strains, including *Escherichia coli* (ATCC 25922), *Pseudomonas aeruginosa* (ATCC 25923), *Bacillus subtillis* (ATCC 11774), *Staphylococcus aureus subsp. aureus* (ATCC 11632), *Aspergillus niger* (439), *Fusarium oxysporum* (M42), *Candida albicans* (ATCC 7754), and *Saccharomyces cerevisiae* (SH 20). The antimicrobial tests were conducted at the Department of Biologically active, Institute of Natural Products Chemistry, VAST.

Positive testing: Streptomycin for Bacteria Gr(+), Tetracyclin for Bacteria Gr(-) and Nystatin or Amphotericin B for Mold and Fungi. Antibiotics mixed in 100% DMSO with appropriate concentration.

Negative testing: Microbiological control does not mix with antibiotics and samples.

Breeding and preservation medium: Saboraud Dextrose Broth (SDB-Sigma) for yeasts and molds. Trypcase Soya Broth (TSB-Sigma) for bacteria.

Experimental medium: Eugon Broth (Difco, USA) for bacteria, Mycophil (Difco, USA) for fungi.

Conduct experiments: The control strains were activated and diluted according to McFarland standard 0.5 and then tested. The plates were incubated at 37 °C/24 h for bacteria and 30 °C/48 h for filamentous fungi and yeasts.

Calculate the result: Minimum inhibitory concentration (MIC-Minimum Inhibitor Concentration) of the sample: Samples were diluted on descending concentration scales, to calculate the minimum inhibitory concentration (MIC), which is the concentration at which the microorganism is almost completely inhibited.

### 3.5. Antioxidant Assays

Dissecting the ability to trap free radicals generated by using DPPH (1,1-diphenyl-2-picrylhydrazyl); Brand-Williams et al. 1995 [19], Shela et al., 2003 [44], Kumar et al., 2013 [45]) is a method that has been recognized for the rapid determination of antioxidant activity. The reagent was dissolved in dimethyl sulfoxide (DMSO 100%) and DPPH was mixed in 96% ethanol. The absorbance of DPPH at λ = 515 nm was determined after adding DPPH to the sample solution on a 96-well microplate. The results are expressed as the mean of at least three replicate trials ± Standard Deviation (*p* < 0.05).

Samples were reconstituted in 100% DMSO at a concentration of 4 mg/mL for the crude extract and 1 mg/mL for the purified sample. Use 5 mM ascorbic acid in 10% DMSO as a positive control. Samples were inoculated onto a 96-well microplate with DPPH solution to obtain final test sample concentrations from 200 μg/mL to 12.5 μg/mL (for crude extracts) and from 50 μg/mL to 3.1 μg/mL (purified sample). Incubate at 37 °C for 30 min and measure Optical Density (OD) at λ = 515 nm on a photometer (Infinite F50, Tecan, Switzerland).

The average value of the ability to neutralize free radicals (Scavenging capacity, SC%) at the individual sample concentrations was entered into the Excel data. The sample (reagent) was diluted to decreasing concentrations and repeated three times at each concentration. The DPPH-induced free radical scavenging efficiency of each sample was calculated based on the percent of free radical neutralization compared to the blank sample (Blank) and negative controls. Samples showing antioxidant activity in the DPPH system were subjected to further steps to find the SC_50_ value (μg/mL, μM/mL). The SC_50_ value is the concentration of the reagent at which 50% of the free radicals are neutralized, determined using the TableCurve v5.0 AISN Software (Jandel Scientific, San Rafael, CA, USA) using the SC% value and a range of similar reagent concentrations. The tests were conducted at the Department of Biologically active, Institute of Natural Products Chemistry, The Vietnam Academy of Science and Technology.

### 3.6. Anti-Inflammatory Assays

The anti-inflammatory activity in-vitro was investigated through the inhibition of NO on RAW264.7 cells (American Type Culture Collection, Manassas, VA, USA) of mice; it was carried out at the Institute of Natural Compound Chemistry, VAST [46]. The RAW264.7 cells (mouse macrophages) were cultured for 48 h in Dulbecco’s culture medium (DMEM-Dulbecco’s Modified Eagle Medium) at 37 °C, 5% CO_2_, 10% Fetal Bovine serum (FBS-Fetal Bovine Serums). The cell fluid was inoculated onto a 96-well microplate, density 2.5 × 10^5^ cells/microplate. Cells were stimulated in 2 µL of the control sample (-) LPS (0.1 mg/mL) for 24 h, supplemented with drugs or reagents at different concentrations with cardamonin (+) as a control. The cell suspension was incubated with Griess reagent and NaNO_2_ at different concentrations to develop a calibration curve. Measuring the reaction mixture at λ = 570 nm. The higher the NO concentration, the higher the optical density, which was determined by the NaNO_2_ standard curve, compared to the % of the control sample as (-) LPS. The ability of the samples to inhibit NO production was determined according to the following as Formula (1):

(1) $$\%\;\mathrm{Inhibitor}=100\;-\;\frac{\mathrm{The}\;\mathrm{concentration}\;\mathrm{of}\;\mathrm{NO}\;\mathrm{sample}}{\mathrm{The}\;\mathrm{concentration}\;\mathrm{of}\;\mathrm{NOLPS}}\times 100$$

## 4. Conclusions

The EOs of aerial parts from four *Asarum* species*: A. geophilum, A. yentunensis, A. splendens* and *A. cordifolium* were obtained by steam distillation and analyzed by GC-MS. The constituents as *α*-pinene (0.33–0.84%), *β*-pinene (0.91–4.71%) and (*E*)*-*nerolidol (0.32–0.79%) were present in all four studied EOs. Sesquiterpene hydrocarbons and oxygenated sesquiterpenes were dominant constituents of *A. geophilum* and *A. splendens* EOs in which 9-*epi*-(*E*)-caryophyllene and eudesm-7(11)-en-4-ol were the major components (18.43% and 13.41%, 15.76% and 14.21%, respectively. Benzenoids were dominant constituents of *A. yentunensis* and *A. cordifolium* EOs, but the major component of *Asarum yentunensis* EO was elemicin (77.20%) while safrole (64.74%) was a major component of *Asarum cordifolium* EO. Especially, the chemical composition of the EOs from *A. yentunensis* and *A. splendens* has been identified for the first time.

The *A. geophilum* EO exhibited good inhibition activity on the *Escherichia coli, Pseudomonas aeruginosa*, *Fusarium oxysporum* and *Saccharomyces cerevisiae* with MIC values of 200 μg/mL. The *A. yentunensis* EO exhibited good inhibition activity on *Escherichia coli, Aspergillus niger* and *Candida albicans* with MIC values of 200 μg/mL, and on the *Staphylococcus aureus* with MIC values of 100 μg/mL. The *A. splendens* EO exhibited good inhibition activity on the *Escherichia coli, Saccharomyces cerevisiae* and *Fusarium oxysporum* with MIC values of 200 μg/mL. The *A. cordifolium* EO exhibited good inhibition activity on the *Escherichia coli, Bacillus subtillis* and *Candida albicans* with MIC values of 200 μg/mL, and on the *Bacillus subtillis* with a MIC value of 100 μg/mL. The *A. geophilum* EO displayed the best antioxidant activity with an SC (%) value of 63.34 ± 1.0%, corresponding to an SC_50_ value of 28.57 µg/mL, following the *A. cordifolium* EO with an SC_50_ value of 57.86 ± 0.8% corresponding to SC_50_ value of 39.62 µg/mL. The *A. splendens* EO displayed the best anti-inflammatory activity with an IC_50_ value of 21.68 µg/mL (corresponding to the inhibition rate of NO production being 69.58 ± 1.3% and the percentage of cell life being 81.85 ± 0.9%).

For the first time, the in vitro antimicrobial, antioxidant and anti-inflammatory activity of the EOs from four *Asarum* species*: A. geophilum, A. yentunensis, A. splendens* and *A. cordifolium* have been studied. They are a very interesting medicinal plant that deserves to be studied for better applications in new therapeutic drugs. However, note that the EOs of *A. cordifolium* and *A. yentunensis* have high amounts of toxic elemicin and safrole, which are potentially toxic when used in overdose.

## Figures and Tables

**Table 1:** Chemical composition (in%) of the EOs of four *Asarum* species.

No.	Chemical Name	Formula		*A. geophilum*			*A. yentunensis*			*A. splendens*			*A. cordifolium*		
			^a^ RI	Time	^b^ RI	%FID	Time	^b^ RI	%FID	Time	^b^ RI	%FID	Time	^b^ RI	%FID
1	*α*-pinene	C_10_H_16_	932	10.01	938	0.33	10.37	939	0.84	10.36	939	0.84	10.70	939	0.56
2	camphene	C_10_H_16_	946	-	-	-	10.87	955	0.39	10.86	955	0.28	11.21	956	0.38
3	*β*-pinene	C_10_H_16_	986	11.36	983	1.45	11.74	984	0.91	11.74	984	4.71	12.11	985	0.96
4	myrcene	C_10_H_16_	988	-	-	-	-	-	-	11.95	991	0.15	12.32	992	0.21
5	*α*-phelandren	C_10_H_16_	1010	12.17	1009	0.13	-	-	-	12.55	1010	0.15	12.93	1010	0.79
6	*δ*-3-carene	C_10_H_16_	1015	-	-	-	12.77	1016	0.39	-	-	-	13.15	1017	0.83
7	*o*-cymene	C_10_H_14_	1022	12.82	1028	0.17	-	-	-	13.20	1029	1.22	13.60	1030	1.80
8	limonene	C_10_H_16_	1030	12.97	1033	0.18	-	-	-	13.36	1034	0.55	13.75	1034	1.87
9	*β*-phelandrene	C_10_H_16_	1031	-	-	-	-	-	-	13.41	1035	0.33	13.82	1036	0.31
10	eucalyptol	C_10_H_18_O	1032	-	-	-	-	-	-	13.48	1037	0.16	-	-	-
11	(*Z*)-*β*-ocimene	C_10_H_16_	1039	-	-	-	13.49	1038	0.13	-	-	-	13.88	1038	1.33
12	(*E*)-*β*-ocimene	C_10_H_16_	1050	-	-	-	13.87	1049	0.42	-	-	-	14.26	1049	0.34
13	terpinolene	C_10_H_16_	1094	15.02	1093	0.20	15.43	1094	0.13	-	-	-			
14	linalool	C_10_H_18_O	1095	15.27	1101	0.18	15.67	1101	3.19	-	-	-	16.10	1102	0.20
15	*exo-*fenchol	C_10_H_18_O	1118	15.95	1120	0.13	-	-	-	-	-	-	-	-	-
16	borneol	C_10_H_18_O	1171	17.84	1174	0.15	18.25	1175	0.31	-	-	-	-	-	-
17	terpinen-4-ol	C_10_H_18_O	1184	18.20	1184	0.30	-	-	-	18.61	1185	0.12	-	-	-
18	*α*-terpineol	C_10_H_18_O	1195	18.65	1197	4.07	-	-	-	19.04	1197	0.26	-	-	-
19	thymol methyl ether	C_11_H_16_O	1232	-	-	-	-	-	-	20.27	1233	0.13	-	-	-
20	geranial	C_10_H_16_O	1271	-	-	-	-	-	-	21.66	1273	0.18	-	-	-
21	bornyl acetate	C_12_H_20_O_2_	1289	21.94	1293	0.23	-	-	-	22.36	1294	1.08	22.85	1295	0.23
22	safrole	C_10_H_10_O_2_	1293	-	-	-	22.59	1300	64.74	-	-	-	-	-	-
23	*n-*tridecane	C_13_H_28_	1300	22.14	1299	0.18	-	-	-	22.56	1299	0.14	-	-	-
24	*δ*-elemene	C_15_H_24_	1343	23.72	1347	0.81	-	-	-	24.16	1348	0.30	-	-	-
25	*α*-terpinyl acetate	C_12_H_20_O_2_	1349	24.00	1355	0.35	-	-	-				-	-	-
26	*endo*-isocamphanyl acetate	C_11_H_18_O_2_	1350	-	-	-	-	-	-	24.43	1356	1.01	-	-	-
27	geranyl acetate	C_12_H_20_O_2_	1379	-	-	-	-	-	-	25.35	1384	0.51	-	-	-
28	*α*-copaene	C_15_H_24_	1394	25.07	1388	0.42	-	-	-	25.53	1389	0.46	26.00	1390	0.50
29	*cis-β*-elemene	C_15_H_24_	1396	25.54	1402	2.75	-	-	-	25.99	1404	3.48	26.45	1404	1.84
30	methyl eugenol	C_11_H_14_O_2_	1403	-	-	-	26.09	1407	1.22	-	-	-	26.58	1408	2.16
31	*β*-caryophyllene	C_15_H_24_	1434	26.60	1436	8.05	27.03	1437	0.29	27.07	1438	9.52			
32	*γ*-elemene	C_15_H_24_	1440	26.84	1444	0.68	-	-	-	-	-	-	-	-	-
33	*α*-*trans-*bergamotene	C_15_H_24_	1443	-	-	-	27.31	1446	0.67	27.32	1446	1.71	-	-	-
34	*β*-gurjunene	C_15_H_24_	1446	27.01	1449	1.66	-	-	-	27.48	1451	4.06	-	-	-
35	aromadendrene	C_15_H_24_	1449	27.20	1455	0.61	-	-	-	27.66	1457	0.60	-	-	-
36	(*Z*)-*β*-farnesene	C_15_H_24_	1451	-	-	-	27.78	1461	2.81	27.79	1461	0.23	-	-	-
37	(*E*)-*β*-farnesene	C_15_H_24_	1460	-	-	-	27.92	1465	0.19	-	-	-	-	-	-
38	*α*-humulene	C_15_H_24_	1471	27.67	1470	3.87	28.11	1471	0.55	28.14	1472	3.72	-	-	-
39	9-*epi*-(*E*)-caryophyllene	C_15_H_24_	1474	27.95	1479	18.43	-	-	-	28.43	1481	15.76	-	-	-
40	*γ-*curcumene	C_15_H_24_	1481	-	-	-	28.66	1488	0.24	-	-	-	-	-	-
41	*γ-*muurolene	C_15_H_24_	1483	-	-	-	-	-	-	28.71	1490	0.59	-	-	-
42	*ar*-curcumene	C_15_H_24_	1484	-	-	-	-	-	-	28.75	1491	0.63	-	-	-
43	germacrene D	C_15_H_24_	1496	28.49	1496	0.56	-	-	-	28.95	1498	1.21	29.44	1499	1.00
44	*n-*pentadecane	C_15_H_32_	1500	28.58	1499	1.10	-	-	-	29.03	1500	0.87	-	-	-
45	(*E*)-methyl isoeugenol	C_11_H_14_O_2_	1501	-	-	-	29.05	1501	0.27	-	-	-	-	-	-
46	*β*-selinene	C_15_H_24_	1502	28.69	1503	0.60	-	-	-	29.16	1505	1.71	29.62	1505	0.17
47	*δ*-selinene	C_15_H_24_	1503	-	-	-	-	-	-	-	-	-	29.65	1507	0.17
48	*trans-*muurola-4(14),5-diene	C_15_H_24_	1503	-	-	-	-	-	-	29.32	1510	0.94	-	-	-
49	viridiflorene	C_15_H_24_	1505	28.93	1511	1.48	-	-	-	-	-	-	-	-	-
50	*γ-*amorphene	C_15_H_24_	1508	-	-	-	-	-	-	-	-	-	29.82	1512	0.33
51	*cis*-bicyclogermacrene	C_15_H_24_	1510	28.98	1512	4.46	-	-	-	-	-	-	29.91	1515	0.73
52	(*E,E*)-*α*-farnesene	C_15_H_24_	1511	-	-	-	29.39	1512	0.36	-	-	-	-	-	-
53	*trans*-bicyclogermacrene	C_15_H_24_	1512	-	-	-	29.42	1513	0.73	29.47	1515	7.50	-	-	-
54	*β*-bisabolene	C_15_H_24_	1514	-	-	-	-	-	-	29.55	1518	1.65	-	-	-
55	*α*-bulnesene	C_15_H_24_	1517	-	-	-	-	-	-	29.64	1521	0.14	30.13	1522	0.20
56	cuparene	C_15_H_22_	1518	-	-	-	-	-	-	29.74	1524	0.16	-	-	-
57	sesquicineole	C_15_H_26_O	1521	-	-	-	29.80	1526	15.34	-	-	-	-	-	-
58	*γ*-cadinene	C_15_H_24_	1524	-	-	-	-	-	-	29.91	1530	0.49	30.44	1533	0.27
59	myristicin	C_11_H_14_O_2_	1529	-	-	-	29.95	1531	0.31	-	-	-	-	-	-
60	*β*-sesquiphellandrene	C_15_H_24_	1531	-	-	-	30.03	1534	0.16	-	-	-	-	-	-
61	*δ*-cadinene	C_15_H_24_	1533	29.65	1535	0.60	-	-	-	30.12	1537	0.72	30.58	1538	0.41
62	zonarene	C_15_H_24_	1537	-	-	-	-	-	-	30.20	1540	0.24	-	-	-
63	*α*-bisabolene	C_15_H_24_	1544	-	-	-	-	-	-	-	-	-	31.00	1552	1.18
64	selina-4(15),7(11)-diene	C_15_H_24_	1545	30.15	1552	1.02	-	-	-	30.61	1553	1.18	-	-	-
65	selina-3,7(11)-diene	C_15_H_24_	1560	30.36	1559	0.41	-	-	-	30.82	1561	1.46	-	-	-
66	elemol	C_15_H_26_O	1562	30.43	1561	0.41	-	-	-	-	-	-	-	-	-
67	elemicin	C_12_H_16_O_2_	1565	-	-	-	-	-	-	-	-	-	31.43	1566	77.20
68	(*E*)*-*nerolidol	C_15_H_26_O	1569	30.64	1568	0.47	31.07	1569	0.41	31.08	1569	0.79	31.60	1572	0.32
69	germacrene B	C_15_H_24_	1572	30.86	1575	0.43	-	-	-	31.33	1578	0.60	-	-	-
70	isoelemicin	C_12_H_16_O_2_	1577	-	-	-	31.33	1578	0.32	-	-	-	-	-	-
71	4-*epi*-maaliol	C_15_H_26_O	1577	31.17	1586	0.67	-	-	-	-	-	-	-	-	-
72	scapanol	C_15_H_26_O	1580	-	-	-	-	-	-	-	-	-	32.32	1596	0.33
73	spathulenol	C_15_H_24_O	1593	31.44	1595	1.24	-	-	-	31.90	1597	1.21	-	-	-
74	viridiflorol	C_15_H_26_O	1595	31.65	1602	2.16	-	-	-	-	-	-	-	-	-
75	caryophyllene oxide	C_15_H_24_O	1599	-	-	-	-	-	-	32.12	1604	1.31	32.65	1607	0.23
76	cubeban-11-ol	C_15_H_26_O	1601	31.91	1611	2.27	-	-	-	-	-	-	-	-	-
77	rosifoliol	C_15_H_26_O	1615	32.14	1620	0.62	-	-	-	-	-	-	-	-	-
78	humulene epoxideII	C_15_H_24_O	1620	-	-	-	-	-	-	32.88	1631	0.14	-	-	-
79	*γ*-eudesmol	C_15_H_26_O	1646	32.96	1648	0.29	-	-	-	33.58	1656	1.31	-	-	-
80	*α*-asarone	C_12_H_16_O_3_	1650	-	-	-	-	-	-	-	-	-	34.09	1658	0.73
81	*α*-muurolol	C_15_H_26_O	1654	-	-	-	-	-	-	-	-	-	34.18	1661	0.24
82	*α*-cadinol	C_15_H_26_O	1665	33.58	1670	0.74	-	-	-	33.64	1658	0.16	34.55	1674	0.84
83	*neo*-intermedeol	C_15_H_26_O	1670	33.67	1674	1.01	-	-	-	-	-	-	-	-	-
84	*β*-asarone	C_12_H_16_O_3_	1678	-	-	-	34.38	1684	0.98	-	-	-	34.88	1686	0.14
85	eudesm-7(11)-en-4-ol	C_15_H_26_O	1709	34.90	1718	13.41	-	-	-	35.40	1721	14.21	-	-	-
86	(*Z*)-ligustilide	C_12_H_14_O_2_	1741	-	-	-	-	-	-	36.29	1755	0.48	-	-	-
87	*n-*tetradecanoic acid	C_14_H_28_O	1759	35.97	1758	0.18	-	-	-	-	-	-	-	-	-
88	isophytol	C_20_H_40_O	1946	40.86	1949	0.12	-	-	-	-	-	-	-	-	-
89	*n-*hexadecanoic acid	C_16_H_32_O_2_	1959	41.17	1962	1.31	-	-	-	-	-	-	-	-	-
90	phytol	C_20_H_40_O	2122	44.81	2117	7.23	-	-	-	-	-	-	-	-	-
91	linoleic acid	C_18_H_32_O	2132	45.30	2139	2.45	-	-	-	-	-	-	-	-	-
92	linolenic acid	C_18_H_32_O	2143	45.44	2145	2.31	-	-	-	-	-	-	-	-	-
Monoterpene hydrocarbons				5 (2.46%)			7 (3.21%)			7 (7.01%)			10 (7.58%)		
Oxigenated monoterpenes				7 (5.41%)			2 (3.5%)			8 (3.45%)			2 (0.43%)		
Sesquiterpene hydrocarbons				17 (46.84%)			9 (6.0%)			25 (59.06%)			11 (6.53%)		
Oxigenated sesquiterpenes				11 (23.29%)			2 (15.75%)			7 (19.13%)			5 (1.96%)		
Derivatives of benzene (benzenoids)				1 (0.17%)			6 (67.84%)			1 (1.22%)			5 (82.03%)		
Others				8 (14.88%)			-			3 (1.49%)			-		
Total				93.05%			96.30%			91.36%			98.78%		

^a^ RIs from NIST Chemistry WebBook. ^b^ RIs relative to n-alkanes (C7–C30) on the HP5-MS column.

**Table 2:** Antimicrobial activity of *Asarum* EOs.

Sample	MIC (μg/mL)							
	EC	PA	BS	SA	AN	FO	SC	CA
*A. geophilum* EO	200	200	-	-	-	200	200	-
*A. yentunensis* EO	200	-	-	100	200	-	-	200
*A. splendens* EO	200	-	-	200	-	200	-	-
*A. cordifolium* EO	200	-	100	-	-	-	-	200

EC: Escherichia coli, PA: Pseudomonas aeruginosa, BS: Bacillus subtillis, SA: Staphylococcus aureus, AN: Aspergillus niger, FO: Fusarium oxysporum, SC: Saccharomyces cerevisiae, CA: Candida albicans.

**Table 3:** Antioxidant activity of genus *Asarum* EOs.

No.	Sample	SC(%) Values	SC_50_ Values (µg/mL)
	Positive control: ascorbic acid	81.55 ± 0.9	13.26
	Negative control [DPPH/EtOH+ DMSO]	0.0 ± 0.0	-
1	*A. geophilum* EO	63.34 ± 1.0	28.57
2	*A. yentunensis* EO	51.58 ± 0.5	50.24
3	*A. splendens* EO	34.24 ± 1.4	>100
4	*A. cordifolium* EO	57.86 ± 0.8	39.62

**Table 4:** Anti-inflammatory activity of genus *Asarum* EOs.

No.	Sample *	The Percentages of Inhibition of NO Production (%)	The Percentages of Cell Life (%)	IC_50_ Values
	Positive control: Cardamonin	85.40 ± 0.7	71.80 ± 0.5	2.33 µM
	Negative control	100.00 ± 0.8	100.99 ± 1.0	-
	LPS	0.00 ± 0.0	-	-
1	*A. geophilum* EO	58.20 ± 0.4	78.05 ± 0.8	40.35 µg/mL
2	*A. yentunensis* EO	53.14 ± 1.6	66.87 ± 1.5	49.87 µg/mL
3	*A. splendens* EO	69.58 ± 1.3	81.85 ± 0.9	21.68 µg/mL
4	*A. cordifolium* EO	40.87 ± 0.6	60.65 ± 1.4	66.37 µg/mL

* The highest concentration of the EO was 100 µg/mL and the refined compounds was 50 µM.

## Data Availability

Not applicable.
